# Commentary: Dairy milk intake and breast cancer risk: does an association exist, and what might be the culprit?

**DOI:** 10.1093/ije/dyaa199

**Published:** 2020-12-17

**Authors:** Aurora Perez-Cornago

In this issue of the *IJE,* Fraser *et al*. report findings on soy and dairy milk intake in relation to breast cancer risk in the Adventist Health Study-2 (AHS-2).^1^ The authors found that soy milk intake was not related to breast cancer risk, whereas higher dairy milk intake was related to a higher risk. This is a well-characterized cohort with a high proportion of vegetarians and vegans (around 36%), making it one of the best cohorts to assess the association between soy intake and breast cancer risk reliably in Western populations. Another related strength of this study is that around 8% of the cohort does not consume dairy milk and there is therefore a large range of intake, which is wider than in most previous prospective studies.^2^

The question of whether dairy milk increases breast cancer risk has been studied for several decades. The latest World Cancer Research Fund meta-analysis concluded that there was no association and found evidence of moderate heterogeneity between studies, which reported inverse, null, and positive associations with breast cancer risk.^2^ The size of the positive association between dairy milk intake and breast cancer risk found in the current AHS-2 study (hazard ratio 1.54, 95% confidence interval 1.24–1.92, for an intake of 316 g/day vs 1.8 g/day)^1^ is large compared with the previous prospective studies.^2^

As the authors highlighted, the observed association of dairy milk consumption with breast cancer risk may be confounded by soya milk consumption;^1^ those in the highest categories of dairy milk consumption consumed less soya milk, and vice versa. This mutual confounding may be an issue particularly in cohorts with high plant milk consumers, and for this reason the authors mutually adjusted for the type of milk in their analyses. Participants who consume no or very little dairy milk, however, may also be different in other ways from those who consume dairy milk; for example, they are likely to be vegans. In the AHS-2, vegans may have a lower risk of breast cancer than non-vegetarians (22% lower risk).^3^ As the authors report in the AHS-2, very low dairy milk consumers are thinner, a factor known to protect against breast cancer risk in postmenopausal women,^2^ who are the majority in this cohort. They are also less likely to participate in screening for breast cancer or to take hormonal therapy for menopause. Although the authors made careful adjustment for all these potential confounders, residual confounding may still operate.

A plausible mechanism which could explain an increase in breast cancer risk with high consumption of dairy milk is circulating concentrations of insulin-like growth factor I (IGF-I). Dairy milk consumers have higher circulating concentrations of IGF-I than non-consumers,^4–6^ and higher circulating IGF-I is associated with higher breast cancer risk, with some genetic evidence suggesting that this relationship is causal.^7^ Interestingly, the authors of the AHS-2 study did not find an association between other dairy products (i.e. cheese and yogurt) and breast cancer risk, although the consumption of cheese and yogurt in the cohort is lower than the consumption of milk.^1^ The largest (>1000 participants) cross-sectional studies on dairy products in relation to IGF-I circulating concentrations have all found a positive association with milk intake,^4–6^ whereas only one has found a positive association with cheese^4^ and yogurt.^5^ The difference of around 2.2 nmol/L in IGF-I concentrations between the highest and the lowest categories of dairy milk intake^4^^,^^6^ would suggest that the higher intake of dairy milk might increase breast cancer risk through IGF-I by around 5%,^7^ which is much smaller than the increase in risk observed in AHS-2; however, there are other growth factors and bovine sex hormones present in dairy products, and it is possible that these and/or other IGFs or their binding proteins also play a role, which might contribute to a larger association with breast cancer risk.

Dairy milk, and particularly its whey fraction,^8^ contains naturally occurring bovine IGF-I, although it is unclear whether the IGF-I in cow’s milk increases blood levels. If so, it is notable that elevated IGF-I concentrations are found in milk from cows treated with recombinant bovine somatotropin [rBST, 60% higher IGF-I than untreated cow’s milk (∼0.3 nmol/L higher)].^9^ The use of rBST in dairy cows by some dairy producers to increase milk production has been a common practice in the USA since the early 1990s,^10^ and over 90% of the participants of the AHS-2 are from this country. However, rBST use was banned in Europe due to concerns about animal health and welfare (e.g. increased risk of mastitis) and inconclusive human health implications.^9^

As well as the possible importance of the bovine IGF-I in dairy milk, other research has suggested that the association between milk intake and blood IGF-I in humans may be due to the proteins and amino acids in dairy milk.^11^ Milk contains two fractions of protein, casein and whey, making up 80% and 20% of milk protein, respectively. It is possible that the stronger association of dairy milk with IGF-I observed in cross-sectional studies and with breast cancer in the AHS-2, compared with other dairy products, may be due to the whey fraction of milk which is lost in some food processing when making different dairy products, such as cheese. Compared with casein, whey protein has a 20% higher content of total branched chain amino acids (BCAA), with over 25% higher content of leucine,^12^ which in turn may stimulate the IGF pathway.^13^

In summary, the wide range of consumption of dairy milk and plant milk alternatives and careful control of confounders in this well-defined cohort make this an important paper on the consumption of different types of milk in relation to breast cancer risk. However, as with all observational studies, the findings may perhaps be influenced by residual confounding and the play of chance. To gain deeper understanding of whether dairy milk may influence cancer risk, more research is needed to clarify whether there are plausible biological mechanisms, including how different dairy products relate to the IGF system, to other hormones and to other bioactive constituents.

## Funding

A.P-C. is supported by a Cancer Research UK Population Research Fellowship (C60192/A28516) and by the World Cancer Research Fund (WCRF UK), as part of the Word Cancer Research Fund International grant programme (2019/1953).

## Conflict of interest

None declared.
